# Detection and pH-Thermal Characterization of Proteinases Exclusive of Honeybee Worker-Fate Larvae (*Apis mellifera* L.)

**DOI:** 10.3390/ijms232415546

**Published:** 2022-12-08

**Authors:** Simona Sagona, Chiara D’Onofrio, Vincenzo Miragliotta, Antonio Felicioli

**Affiliations:** 1 Department of Veterinary Sciences, Pisa University, Viale delle Piagge 2, 56124 Pisa, Italy; 2 Department of Pharmacy, University of Pisa, Via Bonanno 6, 56126 Pisa, Italy; 3 Biosensor Technologies, Austrian Institute of Technology GmbH, Konrad-Lorenz Straße, 24, 3430 Tulln, Austria

**Keywords:** *Apis mellifera*, bee larvae, protease, polyphenism, zymography

## Abstract

The occurrence of the honeybee caste polyphenism arises when a change in diet is transduced into cellular metabolic responses, resulting in a developmental shift mediated by gene expression. The aim of this investigation was to detect and describe the expression profile of water-soluble proteases during the ontogenesis of honeybee worker-fate larvae. The extraction of insect homogenates was followed by the electrophoretic separation of the protein extract in polyacrylamide gels under semi-denaturing condition, precast with gelatin, pollen, or royal jelly protein extracts. The worker-fate honeybee larva showed a proteolytic pattern that varied with aging, and a protease with the highest activity at 72 h after hatching was named PS4. PS4 has a molecular weight of 45 kDa, it remained active until cell sealing, and its enzymatic properties suggest a serine-proteinase nature. To define the process that originates a queen-fate larvae, royal jelly and pollen were analysed, but PS4 was not detected in either of them. The effect of food on the PS4 was investigated by mixing crude extracts of queen and worker-fate larvae with pollen and royal jelly, respectively. Only royal jelly inhibited PS4 in worker-fate larvae. Taken together, our data suggest that PS4 could be involved in caste differentiation.

## 1. Introduction

Polyphenism in *Apis mellifera* is the result of developmental plasticity linked to environmental cues [1]. Caste polyphenism in honeybees occurs when the diet change is transduced into cellular metabolic responses, resulting in a developmental shift mediated by gene expression [1,2,3,4]. Honeybee female larvae are indeed bipotent: they can develop into either a queen or a worker, and the direction of development is determined by diet, starting from the third day of larval life [5]. Within three days after hatching, all the larvae are fed with royal jelly (RJ). Afterward, only the queen-fate larvae continue to receive royal jelly, while the worker-fate larvae are fed a pollen-based diet (worker jelly, WJ) [6,7,8].

The molecular mechanisms connecting diet change to caste polyphenism in the female honeybee is still unclear, although both RJ and WJ chemical composition have been investigated [9,10,11,12,13], and several proteins have been deemed to play a role [14,15,16,17]. Proteolytic activity of proteases, especially serine protease, is a common metabolic feature in both the extracellular digestion of the feed, which is the environmental cue for honeybee caste polyphenism, and the intra-cellular proteolytic activity that may cause polyphenism and metamorphosis [18,19]. As shown in a previous study concerning enzymes involved in different larval instars, chymotryptic proteases were detected in both larvae and adult bees, while tryptic proteases were only detected in adult bees [20]. Furthermore, Chen and colleagues observed that two serine proteases (LOC726352 and LOC409143) had a higher expression in larvae with worker fate than in larvae with queen fate at the fourth and fifth larval instars [21]. Recently, a chymotrypsin-like protease from honeybee larvae was discovered and characterized [22,23], and the presence of two proteases, chymotrypsin-1 and trypsin-1, has also been observed in healthy prepupae [24]. An investigation on proteolytic activity on the body surface of worker- and queen-fate honeybee larvae showed a high enzymatic activity in the former and a total absence of such activity in the latter [25]. Four protein fractions containing active proteases have been detected in the midgut of adult worker bees [26]. The four fractions were trypsin, chymotrypsin, and two endopeptidases, with a molecular mass of 20.5, 19.5, and 30 kDa, respectively (the molecular mass of one of the endopeptidases has not been reported) [26]. In honeybees, larvae protease inhibitors were also detected, specifically, trypsin, chymotrypsin, and cathepsin G inhibitors [27]. The cathepsin G/chymotrypsin inhibitor has also been isolated in drone bee larvae [28]. On the body surface of worker-fate larvae, the presence of protease inhibitors appears high in all seasons, while in the queen-fate larvae it is only present summer [25]. A different expression of proteolytic activity was found during ontogenesis in the solitary bee *Megachile rotundata* [29]. 

The aim of this investigation was to gain insight into honeybee caste polyphenism detecting the differential proteolytic expression patterns in worker- and queen-fate larvae. 

## 2. Results

**Figure 1:**
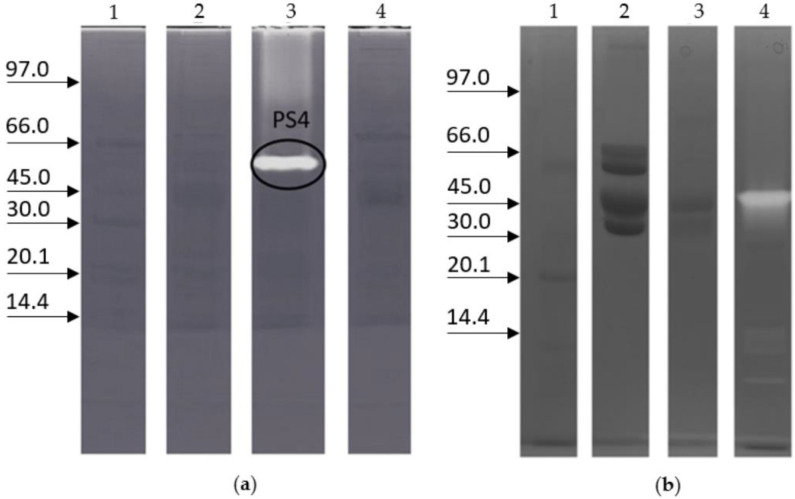
Proteolytic activity in queen-fate larvae and worker-fate larvae of honeybees. (**a**) Zymography (12% T, 2.6% C): lane 1, low marker range in kDa; lane 2, protein extract of queen-fate larvae, 0.15 g; lane 3, protein extract of worker-fate larvae, 0.15 g (5th larval instar), the highlighted band represents the protein PS4; lane 4, protein extract of queen-fate larvae, 0.18 g (5th larval instar). (**b**) Zymography (10% T, 2.6% C): lane 1, marker low range; lane 2, royal jelly extract; lane 3, pollen extract; lane 4, protein extract of worker-fate larvae.

Figure 1a reports the zymography on a 1% gelatine from porcine skin precast gel of protein extracts from worker- and queen-fate larvae of the same weight (lanes 2 and 3) and from worker- and queen-fate larvae both collected on the fifth day after hatching (lanes 3 and 4). The age/weight comparison between the queen-fate larvae and worker-fate larvae was necessary because the two types of larvae have different increases in weight during their development [30]. The investigation aimed to determine whether proteases in queen-fate larvae and worker-fate larvae could vary according to age or weight. In worker-fate larvae extract, the proteolytic activity was observed with at least four proteases and with the molecular weight of one or more proteases ranging from 45–66 kDa. The protease with the highest optical density, hereinafter referred to as PS4, corresponded to about a 45 kDa band (Figure 1a). No proteolytic activity was detected in either queen-fate larval extracts. PS4, the one with the highest activity, was selected for further investigation.

In order to assess if proteolytic activity belonged to RJ or the pollen load of the larval gut, the zymography of the extracts of RJ and pollen was also performed (Figure 1b). The proteolytic activity was present in worker-fate larvae but not in royal jelly and pollen. 

**Figure 2:**
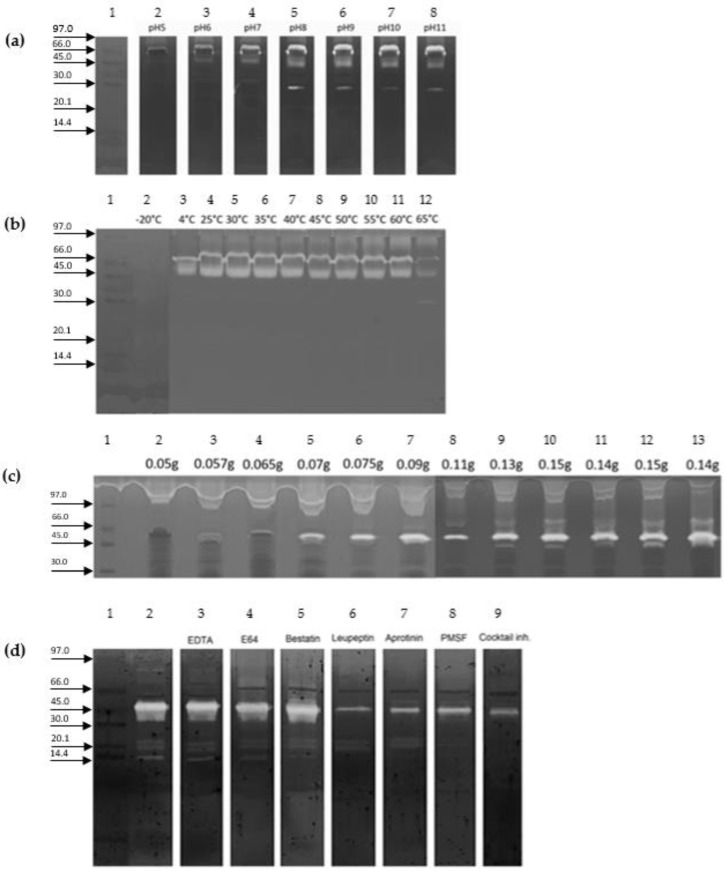
Zymography of worker-fate larvae protein extract (10% T, 2.6% C), marker low range. (**a**) pH activity of PS4: lane 1, Marker low range in kDa; lanes 2–8, worker-fate larval extract incubated with buffer at different pH values (5–11). (**b**) Thermal activity of PS4: lane 1, Marker low range; lanes 2–12, worker-fate larval extract incubated at temperature ranged from −0 °C to 65 °C. (**c**) PS4 activity during worker-fate larvae development: lane 1, Marker low range; lanes 2–10, worker-fate larval extract at different weights (0.05–0.15 g); lanes 11–12, protein extract of sealed worker-fate larvae in the dorsal position with different weights (0.14 g and 0.15 g, respectively); lane 13, protein extract of sealed worker-fate larvae in the ventral position with a weight of 0.14 g. (**d**) The effect of the inhibitor of proteases on PS4: lane 1, marker low range; lane 2, worker-fate larval extract without protease inhibitors; lanes 3–9, worker-fate larval extract incubated with proteases inhibitors.

Densitometry results showed PS4 activity in all the tested pH values with the lowest and highest activities at pH5 and pH10, respectively (Figure 2a). Moreover, PS4 was shown to be active in all the tested temperatures, showing a high activity in the range of temperatures between 25 °C and 60 °C and a low activity at 4 °C and 65 °C (Figure 2b). In Figure 2c, PS4 activity is reported on a porcine gelatine precast gel of crude extracts from different ages of worker-fate larvae, with weights ranging from 0.05 g to 0.15 g. PS4 was shown to be highly expressed in worker-fate larvae from 0.07 to 0.075 g, and its activity did not decrease in prepupae. Leupetin, aprotinin, and PMSF inhibited PS4 at a percentage higher than 50% (Figure 2d). 

**Figure 3:**
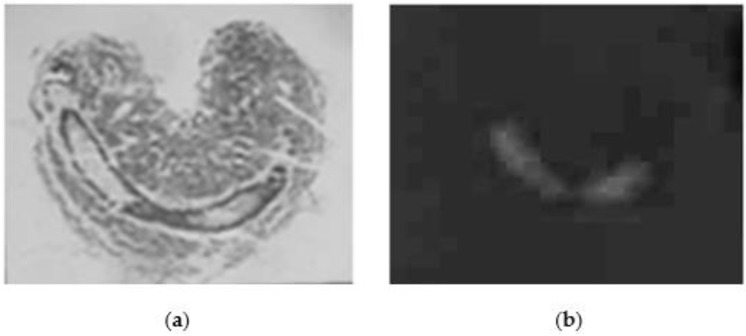
Slide of worker-fate larvae honeybees obtained by cryostat. (**a**) Larva stained by hematoxylin-eosin method; (**b**) larva obtained by zymography treatment; the proteolytic activity is white.

Figure 3 reports both a slide of a larva stained by hematoxylin-eosin method and a slide of worker-fate larva, where the protease activity has been identified in the gut.

**Figure 4:**
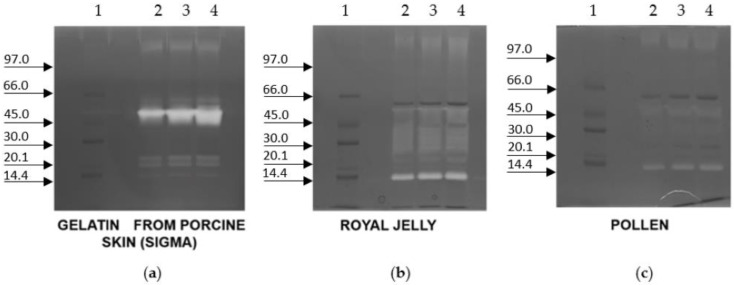
Zymography of the 5th-instar worker-fate larvae crude extract with precast gels containing 0.1% (*w*/*v*) of different substrates: (**a**) gelatine from porcine skin; (**b**) royal jelly; or (**c**) pollen (10% T, 2.6% C). Lane 1, marker low range in kDa; lanes 2–4, different quantities, 10, 15, and 20 µg, of proteins of worker-fate larvae crude extract were loaded in each gel.

Figure 4 reports the zymography of crude extracts from worker-fate larvae on a porcine gelatine, a royal jelly crude extract, or a pollen extract precast gels. Only on the porcine gelatine gel does the PS4 clearly show its activity. On royal jelly crude extract and pollen extract precast gels, a weak signal appeared at the correspondent molecular weight of PS4, suggesting a nonexclusive digestive function of this protease but others have unknown molecular involvements. On the royal jelly crude extract precast gel, another protease with a lower molecular weight than that of PS4 (20 kDa) was detected.

**Figure 5:**
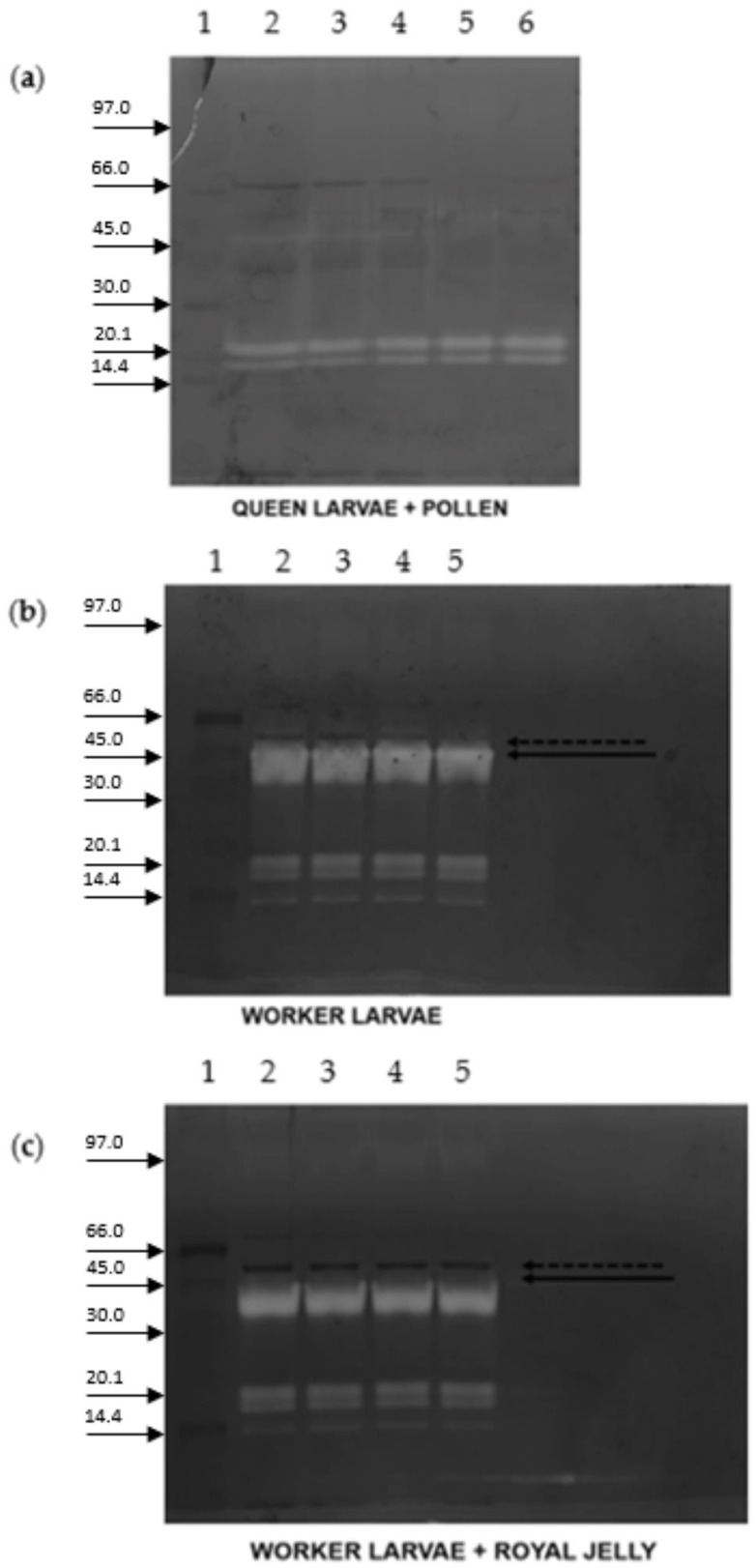
Zymography of the queen-fate larvae protein extracts (10% T, 2.6% C), marker low range. (**a**) Lane 1, marker low range in kDa; lane 2, queen-fate larvae protein extract; lanes 3–6, queen-fate larvae protein extract incubated with pollen crude extract at different times (0, 2, 12, and 24 h). (**b**,**c**) Lane 1, marker low range; lane 2–5, zymography of the worker-fate larva protein extracts incubated without (**b**) or with royal jelly crude extract (**c**) at different times (0, 30, 60, and 120 min). (**b**,**c**) The dotted arrow on the gel indicates the protease named PS4, the solid arrow indicates a new protease.

In order to assess if the pollen or the royal jelly inhibits PS4, queen-fate larvae and worker-fate larvae protein extract were incubated with pollen and RJ, respectively (Figure 5). Queen-fate larvae incubated at different times with pollen crude extract revealed the presence of a protease with a lower molecular weight but the absence of PS4 (Figure 5a). When the worker extract was incubated at different times with royal jelly crude extract, the PS4 was shown to be inhibited, compared with the worker-fate larvae extract where the PS4 was present (Figure 5b,c). 

**Figure 6:**
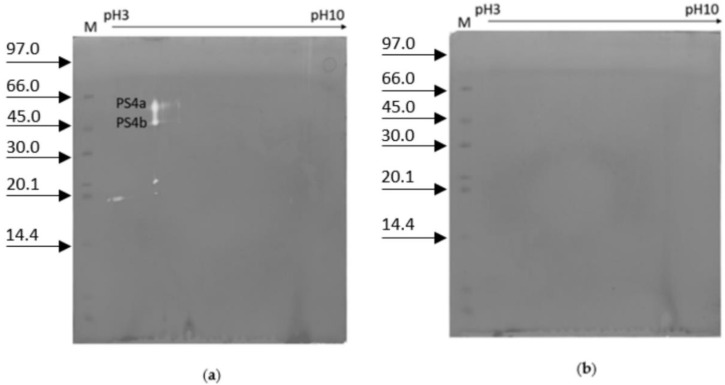
2D zymography of 5th-instar worker-fate larvae crude extract: (**a**) with gut; (**b**) without gut. Strip 11 cm, pH 3–10, T 15%, C 2.6%. M = marker low range in kDa.

In Figure 6a, the 2D zymography of crude extract of worker-fate larvae on the 5th day after hatching is reported. Two spots of proteolytic activity with an apparent isoelectric point of 4.7 and a molecular weight of 57.6 and 49.6 kDa were highlighted, hereinafter referred to as PS4a and PS4b, respectively. In 2D zymography of worker-fate larva without a gut, no proteolytic activity was detectable (Figure 6b).

## 3. Discussion

The absence of detectable proteinase activity in the same age/different weight and in different age/same weight queen- and worker-fate larvae, as well as the absence of PS4 in both pollen and royal jelly samples, suggests that PS4 is a specific worker-fate larvae protein.

The presence of proteases in worker-fate larvae midgut is in accordance with Dahlmann and colleagues, who detected worker-fate larvae proteases, specifically, chymotryptic activity, tryptic activity, and amino-peptidase activity [20]. 

The results obtained in this investigation indicate the presence of at least four proteases. Among them, PS4 was found to be the one with the highest activity; therefore, it was selected for the following investigations. PS4 has a molecular weight of 45 kDa, and it was highly expressed in worker-fate larvae from 0.07 to 0.075 g. This protease was shown to be stable at the different tested pH values (pH range 5–11). Moreover, it was thermostable in a range of temperature between 4 °C and 65 °C, and it was inhibited when stored at −20 °C. In contrast with the results obtained in this investigation, Dahlmann and colleagues reported chymotrypsin A activity in worker-fate larvae gradually decreasing from the larval phase before sealing (about 14%) until the pre-imago instar (<2%), while trypsin activity increased from 10% before sealing to 50% after sealing, when the connection between midgut and hindgut was complete and the indigested feed can pass into the hindgut for the excretion [20]. PS4 resulted in a serine protease since it was inhibited by Leupetin, aprotinin, and PMSF with a percentage higher than 50%. Chen and colleagues reported a higher expression level of two serine proteases in worker-fate larvae, compared to queen-fate larvae [21], giving as an explanation two hypotheses: first, the diet of larvae with worker fate includes royal jelly, pollen, and honey, making it rougher than royal jelly and requiring a stronger digestive ability; second, living in a colony alleviates the pressure on the queen’s immune system. A chymotrypsin-like protease from honeybee larvae has been discovered and characterized, and a role in feed digestion has been hypothesized [22,23].

Matsuoka and co-authors observed in queen-fate larvae the presence of proteases able to digest the royal jelly copolymerized in the gel (carboxypeptidase A-like activity and chymotrypsin-like activity) [31], whereas our results show protease activity in gelatine substrate only in worker-fate larvae. Matsouka and colleagues observed that the optimal condition for chymotrypsin-like activity were 40 °C and pH 9, but they could not detect high inhibition in the range of temperature (20 °C ≤ T ≤ 50 °C) or in the range of pH investigated (4 ≤ pH ≤ 10) [22]. The zymography approach applied on the section of the sagittal plane of the larvae, confirmed by the 2D zymography of worker-fate larva deprived of the gut, revealed that the proteolytic activity was localized in the gut. 

Several authors have observed proteolytic activity in larval food. Rossano and colleagues identified proteolytic activity in the honey, but unfortunately, mass spectrometry analysis did not identify the protease [32]. Erban and colleagues used a proteomic approach to investigate 13 honeys and identified protease activity and three chymotrypsin inhibitors [33]. 

Although trypsin-like protease was found in royal jelly [34], at present, there is not further evidence of proteases in royal jelly [35,36,37] as this study also confirms.

Vinhas and colleagues instead, observed proteases with serine and/or aminopeptidase activity and high molecular weight in pollen [38]. Pollen belonging to different botanical origins was shown to have different activities of trypsin-like, chymotrypsin-like, carboxypeptidase A-like, and carboxypeptidase B-like proteases [39]. Some authors identified other proteases while investigating pollen allergy [40,41]. A 75-kDa peptidase, named acaciain peptidase, was purified and classified as a serine peptidase by Barcia and colleagues [42].

In this work, a zymography of both pollen and royal jelly excluded PS4 belonging to either bee products. 

Since worker-fate larvae are fed with pollen, which is the main protein source [43], and PS4 activity was mainly in the gut, it can be hypothesized that this protease may have a digestive function. Zymography with the copolymerization of pollen or royal jelly in the acrylamide gel excluded digestion as the main function of PS4. 

Pollen has a major role in bees’ health and behavior. It allows the ovarian development of the worker, the intestinal enzyme production in adults, and the development of hypopharyngeal glands [44,45,46]. Crude larval extracts of queen- and worker-fate larvae were co-incubated with pollen and royal jelly, respectively. The crude extract of queen-fate larvae incubated with pollen did not activate PS4, but instead activated other proteases with lower molecular weight. Instead, the crude extract of worker-fate larvae incubated with royal jelly partially inhibited the PS4. Since it is known that the female larval fate is due to the change in diet from royal jelly to pollen on the 3rd day from hatching [6,47], the fact that the royal jelly inhibits the PS4 in worker-fate larvae suggests that this protease could be involved in the caste-differentiation processes. We could speculate that the lack of PS4 inhibition due to the change in the diet could determine the activation of PS4 upstream of a series of unknown molecular events. Further investigation supporting this hypothesis is desirable. In 2D zymography of worker-fate larva on the 5th day, two spots of proteolytic activity were also detected (PS4a and PS4b), confirming that PS4 could consist of two proteases, as suggested by the investigation of royal-jelly inhibition of PS4 in worker-fate larvae. Felicioli et al. observed two proteases, trypsin-1 and chymotrypsin-1, in prepupae [24]. Although these two proteases showed different molecular weight compared with the spots detected in this work, they could correspond to PS4a and PS4b because the semi-denaturing conditions may modify the displacement of proteins in the gel co-polymerized with gelatine. Furthermore, to improve the method for 2D zymography, some modifications have been applied to the protocol of Felicioli et al. [24].

## 4. Conclusions

Previous studies [1,2,3] based on both genetic and molecular approaches contributed to describing the molecular mechanism responsible for the phenotypic plasticity in *Apis mellifera*. The Zymography approach supplies new knowledge in two general areas: the identification of the different genetic expressions in a no-model system and the analysis at the genetic level of the different patterns of genic expression. The knowledge obtained from these two areas allows a genetic study of the polyphenism in different organisms including social insects and other taxa that show big polyphenic expressions. This work suggests that the proteolytic activity might represent a new approach to investigate the polyphenic modification, since the post-translational events caused by proteases often contribute to the different expressions unrelated to the genic differences. Further investigations are desirable in order to clarify the involvement of PS4 in the honeybees’ polyphenism.

## 5. Materials and Methods

### 5.1. Chemicals

Acrylamide and molecular weight marker proteins were purchased from GE Healthcare (Uppsala, Sweden). Coomassie Brillant Blue G-250 was purchased from AppliChem GmbH (Darmstadt, Germany). All other chemicals were purchased from Sigma-Aldrich (St. Louis, MO, USA).

### 5.2. Biological Samples

Worker-fate and queen-fate larvae were collected from June to August from the apiary of the Veterinary Sciences Department of Pisa University (Latitude 43°40′51.45″ N; Longitude 10°20′50.96″ E). In particular, both worker- and queen-fate larvae were sampled at 12, 48, 72, and 96 h post hatching. The post-hatching larval age was assessed by weight [30]. All samples were stored at −20 °C until use. At the same time, in the same apiary, pollen and royal jelly samples were collected for analysis as controls. 

### 5.3. Sample Preparation

Four worker-fate larvae and two queen-fate larvae from the same larval instar were suspended in 50 mM Tris HCl pH 8 buffer and were homogenized by Potter homogenizer. After centrifugation at 19,000× *g* for 60 min at 4 °C, the supernatant was recovered. Pollen-bread and RJ were also used as samples and extracted with the same protocol used for larvae. Protein concentration was determined in accordance with the Bradford method [48] using ovalbumin as a reference. The supernatant was divided into two aliquots stored at −20 °C and at 4 °C until analysis.

### 5.4. Zymography

Zymography was performed in a MiniProtean II apparatus (Bio-Rad, Hercules, CA, USA). Protein extracts underwent proteolytic activity-staining electrophoresis [49,50], using 0.1% porcine gelatine as a precast protein substrate in a discontinuous 10% or 15% T, 2.6% C, Laemmli electrophoresis system under semidenaturing conditions [32]. Before loading, samples were 1:2 diluted with 2% SDS without boiling and in the absence of β-mercaptoethanol. The zymography of the 5th-instar worker-fate larvae crude extract with precast gels containing 0.1% (*w*/*v*) of royal jelly or pollen as a precast protein substrate in a discontinuous 10% T, 2.6% C was also performed.

Aliquots of 40µg of total proteins of the clarified extracts were loaded in each lane. Markers with Low-Range 14.4, 20.1, 30, 45, 66, and 97 kDa were used. After electrophoresis, gels were shaken gently at room temperature for 30 min in 100 mL 2% Triton X-100 in distilled water to remove SDS and restore full enzyme activity.

Gels were then transferred to a bath containing 100 mM Tris- HCl buffer, pH 8.0, and kept with gentle shaking at 37 °C for 2 h and then stained with 0.1% Coomassie Brilliant Blue R-250 and destained with 40% methanol and 10% acetic acid.

For the pH activity assay, electrophoretic slabs of the same crude extract sample were incubated each in 2% Triton X-100 for 30 min, then at 37 °C for 2 h in a 100 mM buffer, following the protocol previously reported by [51]. The buffers were Tris-acetate at pH 5, 6 or 7; Tris- HCl at pH 8 or pH 9; or Borate at pH 10 or 11.

For the thermal stability assay, 10 aliquots of the crude extract of the sample corresponding to the chosen electrophoretic lane were separately incubated for 10 min at −20 °C, 4 °C, 25 °C, 30 °C, 35 °C, 40 °C, 45 °C, 50 °C, 55 °C, 60 °C, or 65 °C prior to the electrophoresis run.

Zymography was also performed using selective inhibitors of proteases: Ethylenediaminetetraacetic acid (EDTA), metalloproteinases inhibitor; E64, cysteine proteinases inhibitor; Bestatin, aminoproteinase inhibitor; Leupeptin, cysteine proteinases and serin-proteinases inhibitor; Aprotinin, serin-proteinase inhibitor; PMSF, serin-proteinases inhibitor; and these proteinases’ cocktail inhibitor (P 9599, SIGMA, St. Louis, MO, USA), [52]. 

### 5.5. 2-DE (Two-Dimensional Gel Electrophoresis) Zymography

Single lanes were densitometrically scanned by Epson Perfection V-750 m Pro scanner and data were analyzed using an Image J software [53]. Protein extract was mixed with a native rehydration solution (Urea 2 M, Chaps 2%, DTT 0.5%, IPG 1%, trace of Bromophenol blue) and loaded on 11 cm strips pH 3–10. Isoelectric focusing electrophoresis was performed at 15 °C on Multiphor II (Amersham Biosciences, Amersham, UK) with the following protocol: 500 V, 1 mA, 5 W, 1 Vh; 500 V, 1 mA, 5 W, 2500 Vh; 3500 V, 1 mA, 5 W, 10,000 Vh; 3500 V, 1 mA, 5 W, 33,250 Vh. The strips were loaded into 15% T, 2.6% C separating polyacrylamide gels with 0.1% porcine gelatine (1 mm thick). SDS–PAGE was performed at 20 mA/gel for 8 min and 30 mA/gel for about 4 h at 8 °C using Hoefer SE 600 Ruby vertical electrophoresis apparatus. After electrophoresis, gels were treated as previously described for zymography. Electrophoretic gels were scanned by Epson Perfection V-750 m Pro scanner and data were analyzed by PDQuest (Bio-Rad, Hercules, CA, USA). 

### 5.6. Histochemistry

In order to investigate the anatomic localization of the proteolytic activity in the larva, worker larvae were sliced by cryostat Leica RM 2055 (Leica Microsystems Srl, Buccinasco (MI), Italy), mounted on 0.1% porcine gelatin on a glass slide, and incubated at 37 °C for 2 h in a 100 mM Tris-HCl buffer. The slide was later stained for zymography as reported. 

Sample sections were also stained with hematoxylin and eosin. The sections were evaluated by light microscopy (Nikon Ni-E, Nikon Instruments, Milan, Italy) for qualitative assessment of morphological features. 

## Data Availability

Not applicable.
